# Immunosuppression Induced by Chronic Inflammation and the Progression to Oral Squamous Cell Carcinoma

**DOI:** 10.1155/2016/5715719

**Published:** 2016-12-08

**Authors:** Yujuan Sun, Nan Liu, Xiaobing Guan, Hongru Wu, Zheng Sun, Hui Zeng

**Affiliations:** ^1^Department of Periodontal & Oral Medicine, Beijing Stomatological Hospital, Capital Medical University, Beijing 100050, China; ^2^Department of Stomatology, Beijing Tongren Hospital, Capital Medical University, Beijing 100005, China; ^3^Institute of Infectious Diseases, Beijing Ditan Hospital, Capital Medical University, Beijing 100015, China; ^4^Beijing Key Laboratory of Emerging Infectious Diseases, Beijing 100015, China

## Abstract

Oral squamous cell carcinoma (OSCC) is an aggressive, invasive malignancy of epithelial origin. The progression from premalignant lesions—oral leukoplakia (OLK) and oral lichen planus (OLP)—to OSCC involves complex inflammatory processes that have not been elucidated. We investigated the roles of inflammatory mediators and infiltrating immunocytes in the pathogenic progression of OLK and OLP to OSCC. The occurrence of regulatory T-cells (Tregs) and tumor-associated macrophages (TAMs) and the expression of anti-inflammatory cytokines and proinflammatory cytokines were investigated in OLK, OLP, and OSCC tissues. Immunohistochemical staining of CD4, FOXP3, CD68, TGF-*β*1, IL-10, IL-4, IFN-*γ*, and MCP-1 showed that the occurrence of Tregs and TAMs increased in parallel with disease progression in OLK and OSCC. IL-10 gradually increased during the early stages of OLK and in OSCC. Infiltrating IL-4^+^ macrophages were seen with increasing frequency in OLK tissue during the progression of oral dysplasia. Fewer TGF-*β*1^+^ macrophages were seen in OSCC than in OLK and OLP. The expression of IFN-*γ* decreased gradually with the OLK development and had the lowest expression in OSCC. MCP-1 expression did not change significantly during the development of OSCC. The results suggested that the immunosuppression induced by chronic inflammation promotes tumorigenesis in OSCC, rather than initiating it.

## 1. Introduction

Numerous epidemiological and molecular biological studies have shown that inflammation greatly increases the risk of cancer. Chronic inflammation can induce persistent tissue damage and changes in the inflammatory cells and cytokines present in the tissue microenvironment. These changes influence the eventual development of tumors, malignant invasion, and metastasis in inflammatory diseases such as ulcerative colitis, atrophic gastritis, Barrett's esophagus, colorectal cancer, and hepatocellular carcinoma [1–6].

The tumor microenvironment includes stromal cells, tumor-infiltrating immune cells, and a range of bioactive substances that suppress the immune response and protect the tumor from immune surveillance. Regulatory T-cells (Tregs) and tumor-associated macrophages (TAMs) comprise the majority of tumor-infiltrating cells associated with immune suppression in the tumor microenvironment [7–9]. The anti-inflammatory cytokines, interleukin-10 (IL-10), IL-4, and transforming growth factor-*β*1 (TGF-*β*1), mediate local immune suppression and promote increased infiltration of Tregs and TAMs. The inflammatory cytokines, interferon-*γ* (IFN-*γ*) and monocyte chemoattractant protein-1 (MCP-1), can either inhibit tumor angiogenesis by inducing cell apoptosis or act as negative regulators of tumor progression. Chronic inflammation may thus influence tumor initiation, progression, invasion, and metastasis via changes in inflammatory-cell populations and cytokine levels in local tissues.

Oral squamous cell carcinoma (OSCC) is the eleventh most prevalent solid tumor worldwide, responsible for approximately 4% of all malignancies [10]. It is an aggressive, invasive epithelial malignancy and is always associated with premalignant oral lesions. Oral leukoplakia (OLK), which appears as “a white area or spot in the oral cavity,” is the most common potentially malignant condition of the oral mucosa [11–13]. Oral lichen planus (OLP) is a chronic T-cell-mediated mucocutaneous inflammatory condition of unknown etiology. It is described by the World Health Organization as potentially precancerous and has been associated with a significantly increased risk of oral cancer [14, 15]. The majority of T-cells in the epithelium affected by OLP and adjacent to damaged basal keratinocytes are activated CD8^+^ lymphocytes, which are known to stimulate apoptosis of keratinocytes. The profile of inflammatory mediators in OLK is also similar to that seen during the progression of OSCC.

The progression from premalignant lesions to OSCC is a chronic, complex, multistep process. The key inflammatory mediator(s) that influence the progression of OLK and OLP to OSCC have not yet been identified. The aim of this study was to further elucidate the role of inflammatory mediators and infiltrating immunocytes in the pathogenesis of OSCC.

## 2. Materials and Methods

### 2.1. Tissue Specimens

Eighty-four specimens, including OLK (*n* = 44), OLP (*n* = 19), and OSCC (*n* = 21), were evaluated by immunohistochemical (IHC) staining. All surgically resected tissue and biopsy specimens were obtained from the Beijing Stomatology Hospital, Beijing, China. OLK specimens were subdivided into three groups, OLK-I (*n* = 20), OLK-II (*n* = 16), and OLK-III (*n* = 8), by the extent of oral dysplasia. The OLP lesions were not treated with steroids. Tissues were fixed in formalin and embedded in paraffin for histopathological examination. Informed consent was obtained from all participants prior to tissue donation. The Ethics Committee of Beijing Stomatology Hospital approved the study protocol. Patient and clinicopathological characteristics are shown in Table 1.

### 2.2. IHC Analysis

The primary antibodies used in the IHC procedures were Foxp3 (1 : 200), IL-10 (1 : 400), IL-4 (1 : 400), IFN-*γ* (1 : 400), MCP-1 (1 : 300; all from Abcam, Cambridge, UK), CD4 (1 : 300, Novocastra, Newcastle, UK), CD68 (KP-1, 1 : 300; DAKO, Glostrup, Denmark), and TGF-*β*1 (1 : 300, Santa Cruz Biotechnology, Texas, USA). All were mouse monoclonal antibodies. Tissue sections were cut from formalin-fixed and paraffin-embedded blocks. Sections were deparaffinized with xylene and then rehydrated through three decreasing concentrations of alcohol. Endogenous peroxidase activity was blocked by incubation in 3% hydrogen peroxide for 10 min. Antigen retrieval was performed by microwaving the samples in 10 mM Tris base, 1 mM EDTA (pH 9.0) buffer. Nonspecific binding was blocked by incubation in 5% bovine serum albumin (GenView, USA) for 20 min at room temperature. Sections were then incubated with the appropriate antibodies at 4°C overnight. Negative control sections were incubated in phosphate buffered saline (PBS). Samples were washed with PBS (pH 7.4) and staining was visualized by incubation with ChenMate EnVision^+^/HRP anti-rabbit/mouse reagent (GeneTech, Shanghai, China) for 30 min at room temperature followed by incubation with 3,3′-diaminobenzidine substrate following the instructions of the assay manufacturer. Stained sections were rinsed gently with distilled water for 10 min, counterstained with hematoxylin and dehydrated through three increasing concentrations of alcohol, cleared in xylene, and mounted on slides. Two pathologists independently counted the number of positively stained cells in five randomly selected fields of each specimen and noted the predominant location(s) within the tissue.

### 2.3. Statistical Analysis

Continuous variables were expressed as the means ± SD, and between-group differences were tested using one-way analysis of variance followed by Scheffé's post hoc test. Pearson's correlation coefficients were calculated and evaluated using Student's *t*-test. Student's *t*-test was used to assess differences between the study groups. A value of *P* < 0.05 was considered significant. The data were analyzed using SPSS software version 13.0 (SPSS Inc., Chicago, IL, USA).

## 3. Results

### 3.1. Distribution of Treg Cells in OLP, OLK, and OSCC

**Figure 1:**
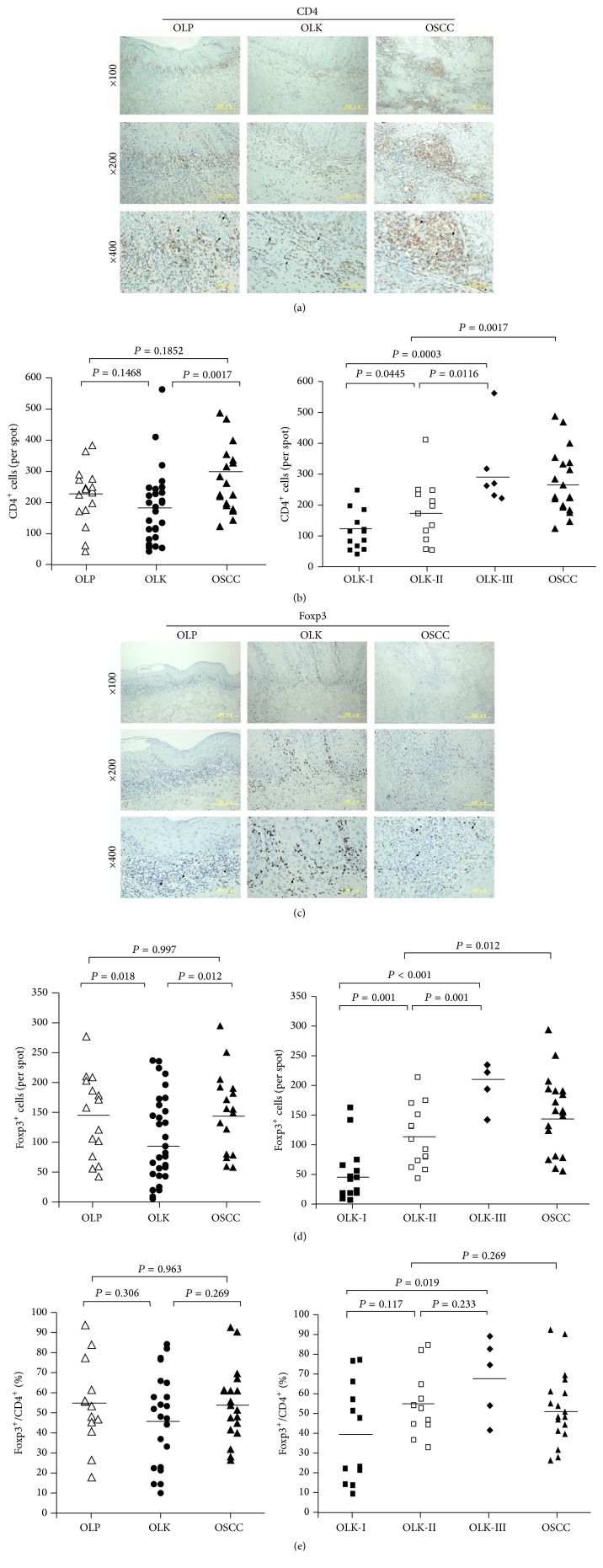
Distribution and frequency of CD4^+^ T-cells and/or Treg cells in OLP, OLK, and OSCC oral tissue samples. (a) Paired CD4- and (c) Foxp3-immunostained sections. (b) Frequency of CD4^+^ T-cells. (d) Frequency of Foxp3^+^ cells. (e) Percentage of Foxp3^+^ Treg-stained CD4^+^ T-cells.

As shown in Figure 1(a), CD4^+^ T-lymphocytes were found predominantly in the superficial lamina propria of OLP and OLK samples or in interstitial tissue of the cancer nests. OLP and OLK contained fewer CD4^+^ T-lymphocytes than OSCC tissues did (Figure 1(b)). The numbers of CD4^+^ T-lymphocytes in OLK-III and OSCC samples were comparable, and both were higher than the numbers seen in OLK-I and OLK-II tissue samples. The IHC results thus suggested that CD4^+^ T-lymphocytes increased gradually with the progression of oral dysplasia (Figure 1(b)).

IHC staining of Foxp3^+^ T-lymphocytes (Tregs) revealed similar distributions of Foxp3^+^ and CD4^+^ lymphocytes in all three groups (Figure 1(c)), but there were significant differences in the number of Foxp3^+^ cells in OSCC compared with OLP or OLK samples (Figure 1(d)). No significant differences in the Foxp3^+^/CD4^+^ ratio were seen in the three groups (Figure 1(e)), but both the number of Foxp3^+^ T-lymphocytes and the Foxp3^+^/CD4^+^ ratio increased gradually with the progression of oral dysplasia in OLK samples (Figures 1(d) and 1(e)).

### 3.2. Distribution of Macrophages in OLP, OLK, and OSCC

**Figure 2:**
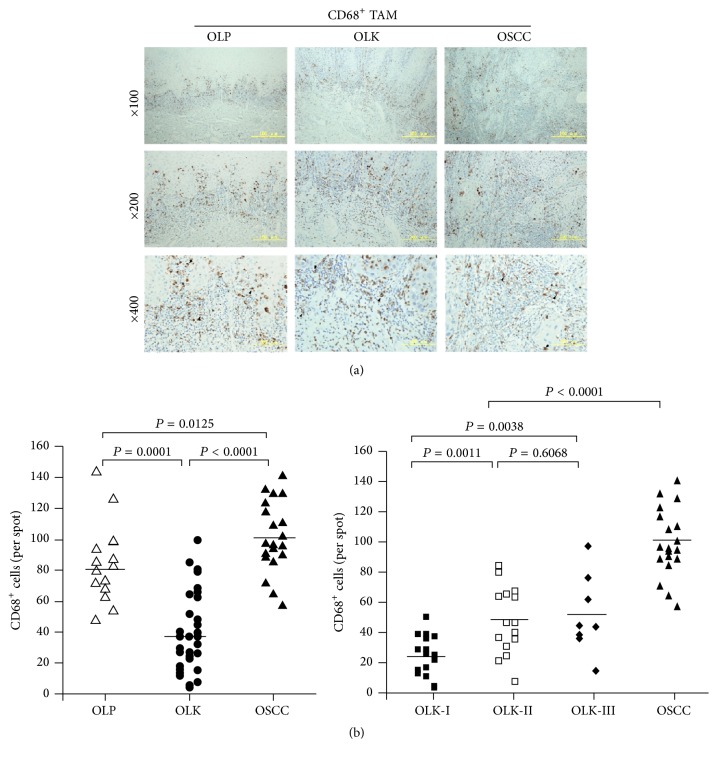
Distribution and proportion of CD68^+^ TAM cells in OLP, OLK, and OSCC tissues. (a) CD68-immunostained sections. (b) Frequency of CD68^+^ TAM cells.

Similar to the pattern seen for CD4^+^ T-lymphocytes, CD68^+^ macrophages (TAMs) were observed predominantly in the superficial lamina propria of OLP and OLK tissues and/or the interstitial tissues of the cancer nests (Figure 2(a)). There were significantly more TAMs in OSCC than in OLK (*P* < 0.0001) and OLP (*P* = 0.0125) tissue. The number of infiltrating TAMs in OLK tissues increased gradually with the progression of dysplasia to OSCC (Figure 2(b)), with maximum infiltration in OSCC tissue (Figure 2(b)).

### 3.3. Changes of Anti-Inflammatory Cytokines (IL-10, IL-4, and TGF-*β*1) in OLP, OLK, and OSCC

**Figure 3:**
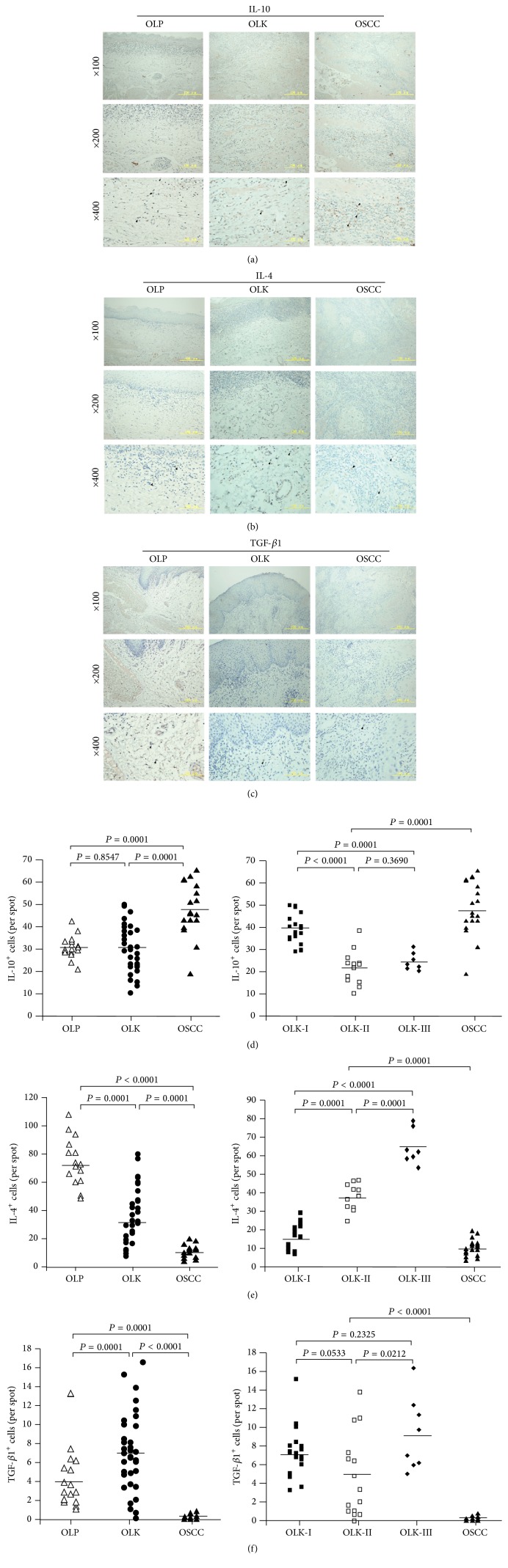
Distribution and frequency of IL-10^+^, IL-4^+^, and TGF-*β*1^+^ macrophages in OLP, OLK, and OSCC tissues. (a) IL-10-immunostained sections. (b) IL-4-immunostained sections. (c) TGF-*β*1-immunostained sections. (d) Frequency of IL-10^+^ macrophages in OLP, OLK-I, OLK-II, OLK-III, and OSCC tissue. (e) Frequency of IL-4^+^ macrophages in OLP, OLK-I, OLK-II, OLK-III, and OSCC tissue. (f) Frequency of TGF-*β*1^+^ macrophages in OLP, OLK-I, OLK-II, OLK-III, and OSCC tissue.

In OLP and OLK tissues, IL-10^+^ and IL-4^+^ macrophages were predominantly detected in the deep lamina propria as well as in all or part of the epithelial layers; cells with positive TGF-*β*1 staining were scattered throughout the lamina propria. In OSCC tissues, IL-10^+^ and IL-4^+^ macrophages were predominantly seen in the cancer nests, and some positive signals were also found in cancerous epithelial tissues (Figures 3(a) and 3(b)). Very few TGF-*β*1^+^ macrophages were seen in OSCC tissues (Figure 3(c)).

There were significantly more IL-10^+^ cells in OSCC than in OLK and OLP tissues (*P* < 0.001, Figure 3(d)), suggesting that IL-10 may contribute to immunosuppression in the cancer microenvironment. However, we did not observe a positive correlation between the number of IL-10^+^ cells and the progression of dysplasia in OLK patients (Figure 3(d)). Significantly fewer IL-4^+^ cells were observed in OSCC than in OLP and OLK tissues, suggesting that IL-4 might not be a key factor for immunosuppression in the cancer microenvironment (Figure 3(e)). However, the number of infiltrating IL-4^+^ macrophages increased gradually with the progression of dysplasia in OLK tissue (Figure 3(e)), suggesting that IL-4 might be a key factor in oncogenesis. In contrast to IL-10, fewer TGF-*β*1^+^ macrophages were seen in OSCC than in OLK and OLP tissues (Figure 3(f)), and there was no correlation between TGF-*β*1^+^ macrophages and the progression of dysplasia in OLK. Thus, TGF-*β*1 might not be a key factor in the development of OSCC.

### 3.4. Changes of Inflammatory Cytokines in OLP, OLK, and OSCC

**Figure 4:**
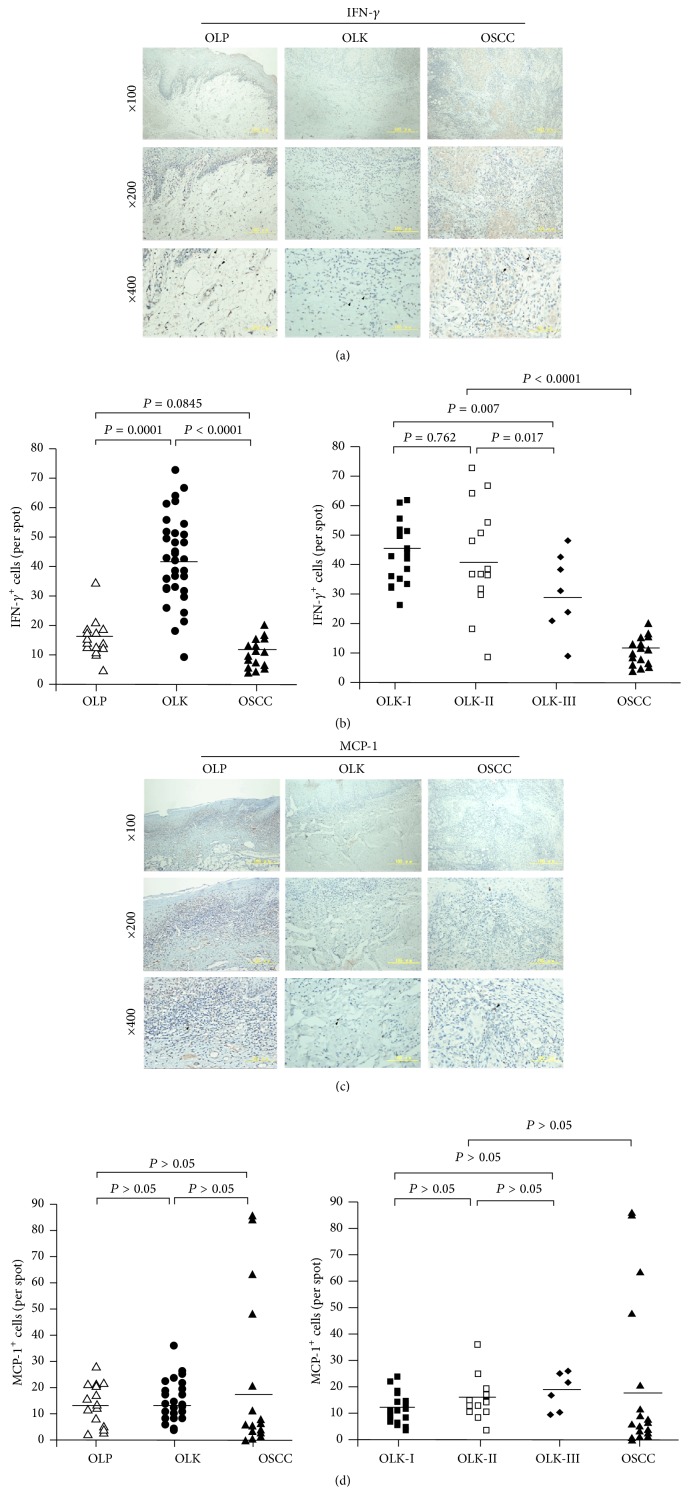
Distribution and frequency of IFN-*γ* and MCP-1 positive macrophages in OLP, OLK, and OSCC tissues. (a) IFN-*γ*-immunostained sections of OLP, OLK, and OSCC tissue. (b) Frequency of IFN-*γ* ^+^ macrophages in OLP, OLK-I, OLK-II, OLK-III, and OSCC tissue. (c) MCP-1-immunostained sections. (d) Frequency of MCP-1^+^ macrophages in OLP, OLK-I, OLK-II, OLK-III, and OSCC tissue.

IFN-*γ* ^+^ macrophages were predominantly detected in OLK; very few were seen in OLP and OSCC tissues (Figure 4(a)), and the number of IFN-*γ* ^+^ cells was negatively correlated with the progression of oral dysplasia in OLK (Figure 4(b)). In OLP, OLK, and OSCC, the distributions of MCP-1^+^ and IFN-*γ* ^+^ macrophages were similar (Figure 4(c)), and no significant differences were observed in the number of MCP-1^+^ macrophages in those tissues (Figure 4(d)). These results suggest that IFN-*γ* ^+^ rather than MCP-1^+^ macrophages are involved in the progression from chronic inflammation to OSCC.

## 4. Discussion

An association between chronic inflammation and tumor progression was proposed nearly 50 years ago [16]. On the one hand, several pathogens, including* Candida*, Epstein-Barr virus (EBV), and human papilloma virus (HPV), have been detected in the oral leukoplakia, which might initiate local inflammatory responses in precancerous lesions [17]. On the other hand, cancer-associated toll-like receptor (e.g., TLR1-4) could be activated by mucosal bacteria, which would cause a carcinogenic process and oncogenic transformation through inducing inflammation [18]. A number of studies published in the past decade have described interactions of solid tumors and the surrounding stroma that contributed to cancer growth, invasion, and metastasis.

The evidence suggests that inflammatory cells and cytokines in the tissues that surround tumors contribute to tumor development and progression in spite of the antitumor responses of the host [19] and that they increase the risk of chronic, inflammatory conditions progressing to cancer. The malignant transformation of OLP and OLK may thus be related to, or dependent on, a series of molecular stimuli originating with inflammatory infiltrate. We investigated key influences in the progression of chronic inflammation to malignant tumors, including tissue-infiltrating immune cells and anti- and proinflammatory cytokines in the OLP, OLK, and OSCC tissue microenvironment.

Treg and TAM cells are key components of the tumor microenvironment, but Foxp3 is not expressed in normal oral mucosa [20]. In keeping with that observation, we found that OLP, OLK, and OSCC tissues contained inflammatory regions that were infiltrated to different extents by CD4^+^ T-cells and CD68^+^ macrophages and that OSCC tissues contained more CD4^+^ T-cells and CD68^+^ macrophages than either OLP or OLK tissue. Moreover, Foxp3, a marker specific for human CD4^+^ CD25^high^ Treg cells [21], was expressed in OLP, OLK, and OSCC tissues. Our data indicate that these immune cells are involved in the regulation of local immune responses. Gradual increases observed in the numbers of infiltrating CD4^+^ T-cells, Foxp3^+^ T-lymphocytes, and CD68^+^ macrophages in parallel with the progression of dysplasia in OLK samples suggested that local changes in the population of immune suppressor cells are an important influence on the malignant progression of tumors. The finding that these immune cells were located close to the basement membrane of the superficial lamina propria in OLK and OLP and in the interstitial tissue of carcinoma nests is consistent with previous reports [22] and suggests a potential possibility of an interaction between these cells.

The infiltration of inflammatory cells during tumorigenesis is accompanied by changes in the local cytokine expression profile. Studies in genetically modified mice and in human tumors found that altered expression of selected pro- (MCP-1, IFN-*γ*) and/or anti-inflammatory cytokines (IL-4, IL-10, and TGF-*β*1) had a crucial role in the promotion of gastric, colorectal, liver, breast, and skin carcinoma development [23]. IL-10 is not expressed in epithelial cells of normal tissues [24, 25], but IHC analysis has demonstrated significantly stronger IL-10^+^ staining of epithelial cell cytoplasm in human oral and pharyngeal carcinomas compared with keratinocytes or inflammatory cells in normal epithelium [26, 27]. Growth of cancer cells is induced by IL-4 via paracrine mechanisms, and the expression of IL-4 has been reported in head and neck squamous cell carcinoma and OSCC [28, 29]. TGF-*β*1 is a negative regulator of cell immune responses and is widely expressed in both normal and tumor cells. However, in squamous cell carcinoma, there is generally a negative correlation between TGF-*β*1 expression and increased dysplasia, with no TGF-*β*1 expression [30].

Interestingly, IL-4, IL-10, and TGF-*β*1 had differing expression in OLP, OLK, and OSCC tissue. OSCC had significantly more IL-10^+^, but not more IL-4^+^ cells, than OLP and OLK tissues, while the number of IL-4^+^, but not IL-10^+^ cells, increased gradually with the progression of dysplasia in OLK tissue. In contrast to the increased numbers of IL-10 and IL-4 in OSCC compared with OLK and OLP tissues, fewer TGF-*β*1^+^ macrophages were detected in OSCC tissues, and no correlation was found between TGF-*β*1^+^ macrophages and the progression of dysplasia in OLK patients. The data thus suggested different roles for these anti-inflammatory cytokines. IL-4 might be important for progression of OLK to a precancerous OSCC condition, but not for the immune evasion of OSCC. IL-10 might not be critical to the development of early-stage malignant dysplasia but contributes to tumor immune escape after progression to OSCC, while TGF-*β*1 might be less important for the development of OSCC.

In addition to anti-inflammatory cytokines, inflammatory cytokines also play an important role in tumor development and progression. IFN-*γ* is an important Th1-type cytokine that is strongly induced by inflammatory stimuli that are also inhibited by IL-4 [31]. In chronic inflammation, M1 macrophages activated by IFN-*γ* have antigen-presenting functions. Th1 immune responses mediated by IFN-*γ* ^+^ macrophages exert antitumor effects and inhibit tumor growth. Infiltration of subepithelial tissue by IFN-*γ* ^+^ cells, including macrophages, contributes to the maintenance of chronic inflammation in OLP [32]. Heimdal et al. [33] found that tumor-infiltrating macrophages were an important source of MCP-1, resulting in recruitment of additional monocytes by positive feedback. In this study, the number of IFN-*γ* ^+^ macrophages decreased with the degree of dysplasia in OLK and was significantly deceased in OSCC. These changes indicate that decreasing expression of IFN-*γ* plays an important role in the progression from OLK to OSCC. MCP-1, a cytokine with a variety of biological activities [34], is secreted into the tumor microenvironment by tumor cells and stromal cells [35]. However, no significant differences in the number of MCP-1^+^ macrophages in OLK, OLP, and OSCC tissues were seen in this study.

Previous studies have shown that interactions of cytokines and/or immune cells are important for the regulation of immune responses [36–43]. We observed increases of CD4^+^ T-cells, Foxp3^+^ T-lymphocytes, CD68^+^ macrophages, and IL-4^+^ cells during the progression of dysplasia in OLK and a parallel decrease in the expression of the inflammatory cytokine IFN-*γ*. These data indicate a shift toward immune suppression during the progression from mucocutaneous inflammatory disease to malignant cancer. Moreover, as IL-4 decreases in OSCC, concomitant increase in IL-10 expression may act to further inhibit immune responses.

## 5. Conclusions

Our study adds to what is known about how chronic inflammation can induce immune suppression and contribute to the occurrence of OSCC. Further studies should be performed to study the inhibition of the development of OSCC by restoring immune homeostasis at the precancerous stage.

## Figures and Tables

**Table 1:** Patient and control characteristics.

	OLP patients	OLK patients	OSCC patients
	(*n* = 19)	(*n* = 44)	(*n* = 21)
*Ages (years) *	51 ± 14	56 ± 12	64 ± 15
*Sex (male/female) *	10/9	25/16	8/13
*Disease stage*			
I/II/III	—	20/16/8	—
*Cancer site*	—	—	
Buccal			2
Floor of mouth			2
Tongue			12
Lip			3
Other			2
*Metastases*	—	—	
No			6
Yes			15

## Abbreviations

CTL: Cytotoxic T-lymphocyte
CTLA-4: Cytotoxic T-lymphocyte-associated antigen-4
Foxp3: Forkhead box P3 transcriptional regulator
IFN-*γ*: Interferon-*γ*
IL-4: Interleukin-4
IL-10: Interleukin-10
MCP-1: Monocyte chemoattractant protein-1
OLK: Oral leukoplakia
OLP: Oral lichen planus
OSCC: Oral squamous cell carcinoma
TAM: Tumor-associated macrophage
TGF-*β*1: Transforming growth factor-*β*1
Treg: Regulatory T-cell.
